# Mid- to long-term outcome of reverse total shoulder arthroplasty as revision procedure for failed hemiarthroplasty after proximal humerus fracture

**DOI:** 10.1186/s12891-024-07870-8

**Published:** 2024-09-20

**Authors:** Alp Paksoy, Doruk Akgün, Jan-Philipp Imiolczyk, Henry Gebauer, Lucca Lacheta, Markus Scheibel, Agahan Hayta, Philipp Moroder

**Affiliations:** 1 Charité University Hospital, Center for Musculoskeletal Surgery, Augustenburger Pl. 1, Berlin, 13353 Germany; 2 Schulthess Klinik, Zurich, Switzerland; 3 University Hospital Rechts der Isar, Technical University Munich, Munich, Germany

**Keywords:** Proximal humerus fracture, Hemiarthroplasty, Revision, Reverse shoulder arthroplasty, Long-term follow-up, Infection, Periprosthetic fracture, Arthroplasty, Prosthesis, Reoperation, Trauma, Grammont reverse prosthesis

## Abstract

**Background:**

Insufficient tuberosity healing is the most common reason for poor outcome after treatment of proximal humerus fractures (PHFs) using hemiarthroplasty (HA). In these cases, revision to reverse total shoulder arthroplasty (RTSA) can improve function and reduce pain in the short term, however, long-term results remain scarce. Aim of this study was to evaluate the clinical and radiological mid- to long-term results in patients with a revision RTSA after failed HA for PHF.

**Methods:**

In this retrospective study all patients that received a revision to RTSA after failed fracture HA between 2006 and 2018 were included. A total of 49 shoulders in 48 patients (38 female, 10 male; mean age 82 ± 9 years) were identified in our database. A total of 20 patients (17 female, 3 male; mean age was 79 ± 9 years) were available for follow-up examination after a mean time period of approximately eight years (3–14 years) after revision surgery. At final follow-up, patients were assessed using a subjective shoulder value (SSV), range of motion (ROM), visual analogue score (VAS), the Constant Score (CS) and the 12-Item Short Form Survey (SF-12).

**Results:**

At final follow-up, mean CS was 55 ± 19 (19–91), VAS averaged 3 ± 3 (0–8) and mean SSV was 61 ± 18% (18–90%). Mean SF-12 was 44 (28–57) with a mean physical component summary (PCS) of 38 (21–56) and a mean mental component summary (MCS) of 51 (29–67). On average active forward flexion (FF) was 104° (10–170°), active abduction (ABD) was 101° (50–170°), active external rotation (ER) was 19° (10–30°) and active internal rotation (IR) of the lumbosacral transition was reached. Three patients presented with a periprosthetic humeral fracture after RTSA implantation and underwent a reoperation (15%) during follow-up period.

**Conclusions:**

Revision RTSA results in promising clinical results in patients after initial failed HA after PHF. A complication and reoperation rate of 15% is tolerable in consideration of satisfactory functional and psychological outcome.

**Trial registration:**

Retrospectively registered.

## Introduction

Proximal humerus fractures (PHFs) are the third most frequent fracture in the elderly following fractures of the proximal femur and the distal radius [1, 2]. Majority of PHFs in the elderly present little displacement and can be successfully managed with conservative treatment [3]. In cases of displaced three- and four-part fractures with a high risk of avascular head necrosis primary arthroplasty has become the most reliable treatment option, as reconstruction is associated with high risk of complication and increased revision rates [4–7].

Despite the decrease in hemiarthroplasty (HA) implantation over the last decade [8, 9], fracture HA remains a treatment option in young patients [10]. Hence, revision strategies for failure are necessary. Causes of unsatisfactory outcomes after HA for PHF treatment are multifactorial [11, 12]. Although satisfactory outcomes can be achieved with HA [13–15], high rate of tuberosity migration [16–21] is a main reason for poor function. Furthermore, patients’ increased age and possible pre-traumatic cuff deficiencies [22] can be responsible for early secondary cuff insufficiency resulting in proximal humeral head migration and poor outcome [18]. Fracture-specific stem design in HA does improve tuberosity fixation and healing which correlates with significantly greater active external rotation (ER), forward flexion (FF) and better functional outcome [23, 24].

To this date, there is no ideal treatment algorithm regarding PHF, however, reverse total shoulder arthroplasty (RTSA) shows superiority over HA in the elderly [5, 18, 25–27]. RTSA has emerged as an attractive option with increasing popularity over the past decade, offering more favorable and predictable functional results (especially in FF and active abduction (ABD)), less residual postoperative pain and allowing early initiation of rehabilitation compared to patients with HA [18, 25, 27–30].

Due to its biomechanical design, RTSA is solely depending on the deltoid muscle. Even in patients with tuberosity migration or insufficiency, good clinical function can be obtained. A meta-analysis for patients with RTSA after acute PHFs show improved ER in those with anatomically healed greater tuberosity, however, its impact on function is not comparable to patients with HA. Although, fracture-specific designs can improve greater tuberosity healing, this does not necessarily correlate with better function [31].

Revision to RTSA is a logical form of treatment, particularly in patients with damaged tuberosities, or rotator cuff pathology [32, 33]. Literature shows that it is a reliable treatment option resulting in stability and providing high potential to improve shoulder function [19]. Although improved clinical short-term results of revision arthroplasty after failed HA have been published [20, 34–36], overall literature and long-term results are scarce. The purpose of this study was to evaluate the mid- to long-term clinical outcome and complications of the revision RTSA after failed HA for PHFs.

## Materials and methods

### Study population

**Fig. 1:**
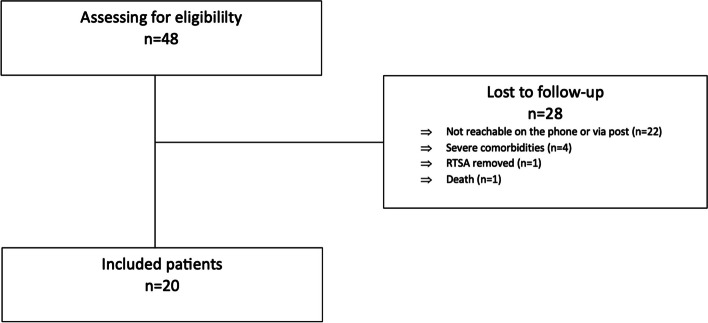
Flow chart of the study participants

Ethical approval from the institutional ethics committee was obtained prior to onset of investigation. Patients who underwent revision surgery for a failed fracture HA to RTSA between 2006 and 2018 were eligible. Inclusion criteria was a revision surgery at our center due to a failed HA after an initially displaced three- and four-part PHF. This resulted in a total of 49 shoulders in 48 patients. The mean age of all patients (38 female, 10 male) was 81.5 ± 8.9 years. A total of 20 shoulders in 20 patients (17 female, 3 male; mean age 79.2 ± 8.8 years) were available for follow-up after a mean period of 7.8 years (3.2–14.4 years). 28 patients were lost to follow-up: One RTSA was explanted at an external hospital, one died, four were not available for in-house examination due to severe medical comorbidities, while 22 patients were not contactable via telephone or post at using their last contact information (Fig. 1).

### Implant design

Given the long follow-up period of this study, a large number of diverse implant designs were included. The implanted prosthetic devices included Global Unite Reverse Fracture (Depuy-Synthes, Warsaw, IN, USA) (*n* = 1), Comprehensive Reverse Shoulder (Biomet, Warsaw, IN, USA) (*n* = 2), Affinis Fracture Inverse (Mathys Ltd., Bettlach, Switzerland) (*n* = 3), Aequalis Reversed Shoulder (Tornier/Stryker Inc., Kalamazoo, MI, USA) (*n* = 23) such as Delta III (*n* = 5) and Delta Xtend (*n* = 15) (DePuy Synthes, Warsaw, IN, USA).

### Surgical technique

All surgeries were performed by one of four specialized shoulder surgeons at our department with each patient in a beach-chair position and under general anesthesia combined with an interscalene block, using the deltopectoral approach. In three cases, the modularity of the stem allowed only the change of the proximal metaphysis. In case of an exchange, the complete stem including the diaphysis was removed. With the sounder in place proximal preparation using metaphyseal punches was performed until the final stem size was established. A humeral retroversion of 20° was preferred; however the retroversion was usually predetermined by the initial fracture stem and initial head osteotomy. A protection plate was placed onto the resection plane during glenoid preparation.

After establishing the initial retroversion of the paleo-glenoid for orientation and size of the baseplate, a central guiding pin was placed via the template, to allow for correct glenoid reaming. Whenever necessary, due to eccentric glenoid erosion, high-side reaming was performed to establish a plane glenoid surface allowing baseplate fixation. The glenoid components were assembled and implanted according to the manufacturer’s technical manual. The glenosphere diameter was selected based on the surgeon’s preference, joint stability and tuberosity reduction. In none of the cases, it was necessary to use any type of supplementation in the glenoid.

The humeral resection plate was removed, and the definitive stem was placed using the compactor before the definitive tray was placed. In cases of intraoperative humerus fractures during stem removal, additional metal and/or polymer cables were placed above and below the location of fracture line. In all cases, wherever conversion was not possible and rotational stem stability was not achieved with trial implants, hybrid cementation of the diaphyseal stem component was performed (*n* = 18). After joint reduction, subscapularis repair and wound closure were performed. In addition, fixation of greater tuberosity and posterior cuff was performed whenever possible. In most cases, there were no tuberosities remaining due to resorption after mal- or non-union following HA implantation.

Revision surgeries with single- (*n* = 3) or two-stage (*n* = 3) exchange were exclusively performed at our institution, following a standardized revision arthroplasty protocol. Patients with signs for high-grade infections like acute symptoms of local inflammation and/or sinus tract with evidence of communication to the joint or visualization of the prosthesis, or poor soft tissue were admitted to the two-stage revision. The routine PJI protocol at our institution included laboratory values such as serum CRP levels, leucocyte counts, and the proportion of polymorphonuclear leucocytes in aspiration, as well as microbiological and histopathological findings at the time of revision surgery.

In cases of one- or two stage-revision, all hardware, sutures, cement, and infected tissue were removed. Scar formations were accurately dissected, incorporating a careful release and mobilization of the remaining rotator cuff. This was followed by meticulous irrigation with high-pressure pulsatile lavage, debridement, and the implantation of a custom made, antibiotic-loaded cement spacer with a consistent utilization of the deltopectoral approach in all cases. In cases of one stage-revision, the implantation of the RTSA was performed according to the details described above. After the initial stage, an antibiotic regimen, recommended by our infectious diseases department based on the current literature and experience of our department, was administered postoperatively to all patients [37–40].

In cases of two-stage revision, the reimplantation was carried out via the same deltopectoral approach. This operation also served as another opportunity for comprehensive debridement of surrounding soft tissues and bone, preceding the reimplantation of definitive components. After the second stage, a tailored intravenous antibiotic treatment was administered for one to two weeks, followed by transitioning to an oral regime to complete a total treatment duration of a minimum of six weeks after reimplantation.

### Rehabilitation protocol

Patients followed a standardized postoperative rehabilitation protocol. The operated shoulder was immobilized in a sling in internal rotation for six weeks. During this time, only supervised, passive mobilization (excluding external rotation exceeding 0°) was performed during physiotherapy sessions. After six weeks, pain-free active motion was added to the protocol. Strengthening exercises were carefully introduced twelve weeks postoperatively.

### Clinical and radiographic evaluation

Clinical outcome was assessed using Constant-Murley score (CS) [41], including its Visual Analogue Score (VAS) for pain and patient-reported Subjective Shoulder Value (SSV) [42] at follow-up examination. Moreover, the 12-Item Short Form Survey (SF-12) [43] was obtained, which consists of the physical (PCS) and mental component summary (MCS).

Active range of motion (ROM) was documented for ABD, FF, ER, and internal rotation (IR). ER was assessed standing with arm at side, IR was categorized by the ability to reach different levels of the spine with the thumb. Postoperative complications were documented throughout the follow-up period. The standard diagnostic protocol was established using institutional criteria according to European Bone and Joint Infection Society to identify periprosthetic joint infection (PJI) [44].

Patients were assessed on standardized true anteroposterior, axillary, and Y-view radiographs to identify implant loosening (in terms of subsidence or shift in position), implant breakage, greater tuberosity insufficiency, and heterotopic ossification [45]. Scapular notching was evaluated according to Sirveaux classification [46].

### Data management and statistical analysis

All data were recorded pseudonymously regarding reason for revision, type of implant, operated shoulder, surgeon, time interval between primary HA implantation and revision to RTSA as well as further demographic data (gender, age etc.). All functional outcome measurements were recorded. All intra- and postoperative adverse events were documented. The rate of complication and reoperation were calculated for this respective patient cohort.

A descriptive analysis of the variables was performed by calculating mean, median, standard deviation (±), absolute and percentage frequency, for which IBM SPSS Statistics 29.0 software (IBM, Armonk, NY, USA) was employed. The two-sample t-test (for parametric distribution) or Mann-Whitney-U test (for non-parametric distribution) was used to compare continuous variables between groups. We have performed two subgroup analysis for initial reason of HA revision and divided by timing of RTSA revision after HA implantation (cut-off: two years). Statistical significance level was set to 0.05.

## Results

All clinical results of these patients are illustrated in Table 1 and their demographics are summarized in Table 2.

**Table 1:** Detailed illustration of every patient included showing baseline demographics, implant design, glenosphere size, follow-up results, and complication. F, female; M, male; r, right; l, left; GS, Glenosphere size; SSV, subjective shoulder value; VAS, visual analogue score; CS, constant score; SF-12, 12-Item short form survey; IR, active internal rotation (0: lateral side of the thigh, 2: buttocks, 4: lumbosacral transition, 6: third lumbar vertebral body, 8: 12th thoracic vertebral body, 10: between shoulder blades); ER, active external rotation; FF, active forward flexion; ABD, active abduction; con, conversion; Ex, total exchange; tub. insuf., tuberosity insufficiency; PJI, periprosthetic joint infection; perip. fx, periprosthetic fracture

Sex (F/M)	Side (*r*/l)	Age (years)	Prothesis type	GS (mm)	Follow-up (years)	SSV (%)	VAS (points)	CS (points)	SF-12 (points)	IR (points)	ER (°)	FF (°)	ABD (°)	Type of revision from HA (Con/Ex)	Complication after HA	Complication after RTSA
**F**	r	87	Delta III	42	14.4	80	2	53	49	2	30	80	30	Ex	tub. insuf.	-
**F**	r	76	Delta Xtend	42	13.1	60	0	51	33	4	20	90	20	Ex	PJI	-
**M**	r	71	Delta Xtend	42	11.9	75	0	80	45	6	30	170	30	Ex	tub. insuf.	-
**M**	r	80	Aequalis	36	8.3	80	0	73	57	2	30	170	30	Ex	PJI	-
**F**	r	86	Aequalis	36	7.6	50	0	46	44	2	10	80	10	Con	tub. insuf.	-
**F**	r	86	Aequalis	36	7.3	80	5	38	28	4	20	70	20	Ex	PJI	-
**F**	l	79	Aequalis	36	7.2	20	8	19	33	4	10	50	10	Ex	PJI	perip. fx
**F**	r	75	Aequalis	42	5.6	50	0	42	45	2	10	50	10	Ex	PJI	-
**F**	r	56	Aequalis	36	5.3	60	4	72	57	4	25	165	25	Con	tub. insuf.	-
**F**	r	79	Delta Xtend	38	13.3	90	0	75	54	0	30	115	30	Ex	tub. insuf.	-
**F**	r	82	Affinis	39	12.4	60	2	46	42	6	30	115	30	Con	tub. insuf.	-
**F**	l	78	Delta Xtend	38	11.9	70	5	38	39	2	10	85	10	Ex	tub. insuf.	-
**F**	l	97	Aequalis	36	8.4	50	0	40	48	4	20	70	20	Ex	tub. insuf.	-
**F**	r	88	Aequalis	36	7.1	50	5	35	44	2	10	70	10	Ex	PJI	perip. fx
**F**	r	66	Aequalis	36	3.5	50	5	56	46	0	10	110	10	Ex	tub. insuf.	-
**M**	r	81	Aequalis	36	4.1	60	4	68	42	6	10	80	10	Ex	tub. insuf.	-
**F**	l	77	Delta Xtend	38	3.2	50	2	80	45	6	20	130	20	Ex	tub. insuf.	-
**F**	r	72	Global Unite	42	4.4	90	0	91	54	8	30	170	30	Ex	tub. insuf.	-
**F**	r	83	Aequalis	36	4.1	30	7	32	38	2	10	70	10	Ex	tub. insuf.	perip. fx
**F**	l	86	Aequalis	36	3.4	60	3	54	46	6	10	70	10	Ex	tub. insuf.	-

**Table 2:** Patient demographics. Values of age at follow-up and follow-up period are reported as means with standard deviations (±) (ranges). Total number of patients: 20

Patient demographics	
Sex (female/male) (n)	17/3
Operated side (right/left) (n)	15/5
Age at follow-up (years)	79.2 ± 8.8
Follow-up period (years)	7.8 (3.2–14.4)

**Fig. 2:**
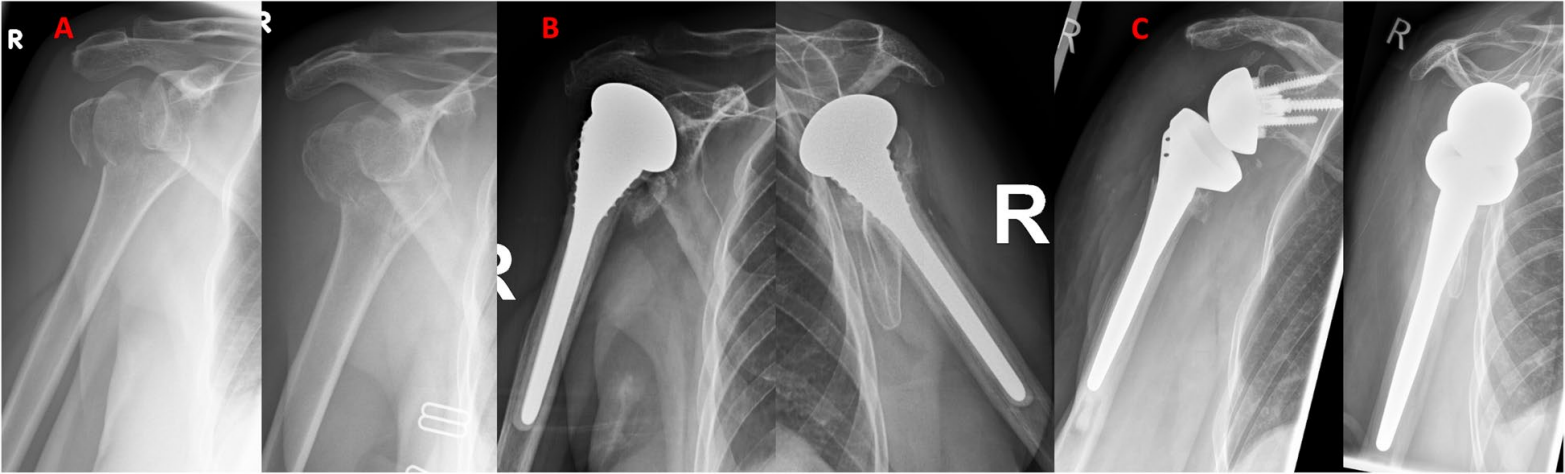
Radiographs obtained during the course of reverse total shoulder arthroplasty revision procedure for failed hemiarthroplasty after proximal humerus fracture. **A** A 66-year-old patient from our cohort fell on to her right shoulder at home, hemiarthroplasty was indicated in this case. **B** Prosthesis failure due to tuberosity and rotator cuff insufficiency with postoperative persistent pain and stiffness. **C** Implantation of a reverse total shoulder endoprosthesis was performed

 The most frequent indication for revision surgery was rotator cuff insufficiency due to tuberosity insufficiency (*n* = 32, Fig. 2), followed by acute postoperative infection (*n* = 11), instability (*n* = 4), periprosthetic fracture (*n* = 1), and poor function due to incorrect implant positioning (*n* = 1). The mean time interval between the primary fracture HA and the revision surgery to RTSA was 2.6 years (0.1–12.8 years) for all patients.

The most common used prosthetic device included the Aequalis Reversed Shoulder (Tornier/Stryker Inc., Kalamazoo, MI, USA) in 12 cases (60%) and the most common indication for revision surgery was rotator cuff deficiency due to greater tuberosity insufficiency in 14 patients (70%). Only in three cases a conversion without the exchange of the well-fixed stem was possible. In five cases (25%), an intraoperative humerus fracture occurred during stem removal, that needed additional cable wire fixation. In all six cases with PJI, acute deep infections were observed following the first six months after HA implantation. Hence, complete implant removal and radical debridement was performed to eradicate infection performing a single- (*n* = 3) or two-stage (*n* = 3) revision. No recurrent infections in these revision cases were observed at the final follow-up. In the remaining eleven patients (55%), a complete exchange was necessary because the implanted HA stem design did not allow RTSA conversion.

### Clinical and radiographic outcomes

Clinical outcome of patients eligible for follow-up examination are summarized in Table 3. There were no cases of osteolysis around the baseplate or fixation screws reported in our cohort. There was one single case of humeral stem loosening (5%). There were five cases with glenoidal heterotopic ossification and two cases with humeral heterotopic ossification, whereas no signs of heterotopic ossification were observed in 13 cases. Seven patients demonstrated scapular notching of grade one (35%), one patient with grade 2 notching (5%), while twelve patients had no scapular notching. There was one case of greater tuberosity resorption, in all other 19 cases, greater tuberosity was resorbed at time of revision RTSA implantation.

**Table 3:** Clinical outcome measurements. Values reported with standard deviations (± SD) (ranges). SSV, subjective shoulder value; VAS, visual analogue score; CS, constant score; SF-12, 12-Item short form survey; PCS, physical component summary; MCS, mental component summary; FF, active forward flexion; ABD, active abduction; ER, active external rotation; IR, active internal rotation (lateral side of the thigh, buttocks, lumbosacral transition, third lumbar vertebral body, 12th thoracic vertebral body, between shoulder blades)

Clinical Outcome	Value
SSV (%)	60.8 ± 18.4 (18.4–90.0)
VAS (points)	2.6 ± 2.6 (0–8)
CS (%)	54.5 ± 19.3 (19–91)
SF-12 (points)	44 (28–57)
PCS (points)	38 (21–56)
MCS (points)	51 (29–67)
FF (°)	104 ± 44 (10–170)
ABD (°)	101 ± 41 (50–170)
ER (°)	19 ± 9 (10–30)
IR (points)	3.5 ± 2.0 (0–6)

### Complications

Three patients presented with complication that needed revision surgery due to periprosthetic humerus fractures after RTSA implantation following a fall onto the operated shoulder. One periprosthetic fracture needed stem revision after 12 years and additional cable wire fixation, whereas the other two were treated with open reduction and internal fixation using a locking plate three months and twelve years after RTSA implantation. Other complications, such as infections, phlebitis, neurological issues, intraoperative fractures of the glenoid and acromion, stress fractures, and muscular complications, were not observed in the patients eligible for follow-up examination.

### Subgroup analysis

There was no statistically significant difference between the subgroups early (< 2 years) and late (≥ 2 years) RTSA after fracture HA (Table 4). Patients who were revised to RTSA after fracture HA due to tuberosity insufficiency showed better outcomes than those with revision due to PJI in terms of PCS (38.5 ± 10.8 vs. 36.1 ± 10.7, respectively; *p* < 0.05), MCS (53.9 ± 7.8 vs. 43.5 ± 13.4, respectively; *p* < 0.05) and FF (109.6 ± 44.4 vs. 90.0 ± 44.3, respectively; *p* < 0.05) (Table 5). A trend that is not statistically significant was encountered in VAS: The group of patients with revision to RTSA after fracture HA due to tuberosity insufficiency showed less postoperative pain than those with revision due to PJI (2.4 ± 2.3 vs. 3.0 ± 3.5, respectively, *p* = 0.070) (Table 5).

**Table 4:** Subgroup analysis of clinical outcome between patients that received revision to reverse total shoulder arthroplasty at an early (within first two years) or late stage (after two years) after fracture hemiarthroplasty. Values reported as means with standard deviations (±) (ranges). PJI, periprosthetic joint infection; SSV, subjective shoulder value; VAS, visual analogue score; CS, constant score; SF-12, 12-Item short form survey; PCS, physical component summary; MCS, mental component summary; FF, active forward flexion; ABD, active abduction; ER, active external rotation; IR, active internal rotation (lateral side of the thigh, buttocks, lumbosacral transition, third lumbar vertebral body, 12th thoracic vertebral body, between shoulder blades)

Value Clinical Outcome	Early (*n* = 11; <2 years)	Late (*n* = 9; ≥2 years)	*p*-value
SSV (%)	57.7 ± 18.9	64.4 ± 18.1	0.991
VAS (points)	2.2 ± 2.9	3.1 ± 2.4	0.389
CS (points)	57.0 ± 20.6	50.7 ± 18.4	0.424
SF-12 (points)	45.8 ± 8.0	42.4 ± 7.5	0.573
PCS (points)	40.8 ± 8.9	34.1 ± 11.7	0.372
MCS (points)	50.7 ± 11.6	50.8 ± 10.0	0.865
FF (°)	109.5 ± 51.4	96.7 ± 35.01	0.387
ABD (°)	110.9 ± 43.3	87.8 ± 35.4	0.259
ER (°)	19.5 ± 8.5	17.7 ± 9.7	0.292
IR (points)	3.1 ± 2.1	4.3 ± 2.5	0.347

**Table 5:** Subgroup analysis of clinical outcome comparing reasons for reverse total shoulder arthroplasty revision (periprosthetic joint infection and tuberosity insufficiency). Values reported as means with standard deviations (±) (ranges). PJI, periprosthetic joint infection; SSV, subjective shoulder value; VAS, visual analogue score; CS, constant score; SF-12, 12-Item short form survey; PCS, physical component summary; MCS, mental component summary; FF, active forward flexion; ABD, active abduction; ER, active external rotation; IR, active internal rotation (lateral side of the thigh, buttocks, lumbosacral transition, third lumbar vertebral body, 12th thoracic vertebral body, between shoulder blades)

Value Clinical Outcome	PJI (*n* = 6)	Tuberosity Insufficiency (*n* = 14)	*p*-value
SSV (%)	56.7 ± 22.5	62.5 ± 17.0	0.526
VAS (points)	3.0 ± 3.5	2.4 ± 2.3	**0.070**
CS (points)	43.0 ± 18.1	58.9 ± 18.5	0.456
SF-12 (points)	39.8 ± 10.6	46.2 ± 5.6	0.607
PCS (points)	36.1 ± 10.7	38.5 ± 10.8	**0.010**
MCS (points)	43.5 ± 13.4	53.9 ± 7.8	**0.036**
FF (°)	90.0 ± 44.3	109.6 ± 44.4	**0.042**
ABD (°)	83.3 ± 45.0	107.9 ± 38.0	0.996
ER (°)	16.7 ± 8.2	19.6 ± 9.3	0.977
IR (points)	3.0 ± 1.1	3.9 ± 2.7	0.375

## Discussion

Our results demonstrate that patients achieve acceptable clinical outcome after revision RTSA for failed HA fracture treatment. After a mean follow-up of eight years, mean SSV score was 61% and mean CS averaged 55 points, indicating good and reasonable function. Complication and reoperation rates of 15% after recurrent falls are tolerable in this elderly population. Although mean SF-12 score of 44 points does not indicate an outstanding outcome with a relatively low mean PCS of 38 points, a mean MCS of 51 points was reached.

Patients treated with RTSA for PJI showed trends towards worse clinical outcome with regards to PCS, MCS, FF, and VAS, compared to patients who were revised to RTSA after fracture HA due to tuberosity insufficiency. In cases of acute PJI, meticulous operative debridement with implant retention followed by antibiotics are mandatory in combination with a single- or two-stage revision. In the present study, patients with tuberosity insufficiency illustrated overall better clinical outcome and less pain resulting in higher levels of mental health and subjective outcome, compared to patients treated with RTSA for PJI.

Patients who were revised relatively early to RTSA showed similar results with no statistically significant differences compared to patients with longer HA survival time (cut-off at two years after initial HA surgery). One could expect that early problems such as postoperative pseudoparalysis due to early tuberosity migration, or early loosening and pain due to high virulent pathogens that lead to early revision may have influenced the surrounding tissue and therefore impact outcome of RTSA. Patients with late revisions had an initial successful treatment with HA and therefore good function and cuff status. Late revisions caused by secondary rotator cuff deficiency or glenoid bone erosion can be successfully treated with RTSA due to its design. Nevertheless, no differences were shown between patients with initially successful HA treatment and late revision and patients with early revision. One could argue that HA can be used as a treatment option in young patients with satisfactory bone quality, intact rotator cuff and good healing biology, postponing RTSA implantation.

Regarding the good performance in MCS with 51 points, clinical results of our study were evaluated as satisfactory. Mean SF-12 score in our follow-up group was 44 points, which at first did not indicate an ideal outcome with a relatively low mean PCS of 38 points. However, SF-12 consists of two summary measures, PCS and MCS, and in detail a subset of 12 items from SF-36 Health Survey [43, 47, 48] covering the same eight domains of health outcomes, including physical functioning, role-physical, bodily pain, general health, vitality, social functioning, role-emotional, and mental health. In an elderly population with a failed HA, pain relief and necessary improvement of ROM mainly influence patient satisfaction in a positive way due to low age-appropriate demands of activities of daily living, as opposed to achieving maximum ROM, which presumably did reflect in the discrepancy of our mean physical and psychological scores of the SF-12.

Available literature shows similar clinical results to the presented study in terms of CS, VAS, complication rate and ROM. Holschen et al. included 44 failed shoulder arthroplasties converted to RTSA in most of the cases with Delta Xtend prosthesis (DePuy Synthes, Warsaw, IN, USA) (*n* = 31; 70.5%) from 2010 to 2012 with a follow-up of 24 months (14–36 months) [19]. Only 21 of 43 patients were treated for PHF [19]. Patients revised because of failed fracture arthroplasty illustrated a CS of 55 points, VAS of 2.2, complication rate of 24%, FF of 115°, and ABD of 107° [19]. Gohlke et al. designed a similar study from 2000 to 2005 with 84 shoulder replacement revisions (Tornier/Stryker Inc., Kala-mazoo, MI, USA), of which 34 were revisions of failed fracture HA with an average follow-up of 31.5 months (12–59 months) [35]. At final follow-up, patients showed CS of 45 points, complication rate of 24%, FF of 125° and ABD of 95° [35]. The study conducted by Reuther et al. between 2008 and 2015 with a mean follow-up period of 55.1 months (12.0-91.1 months) had 17 patients with Affinis Inverse or Affinis Facture Inverse system (Mathys, Switzerland) implanted as primary prosthesis and without any stem removal during revision for failed HA after PHF [49]. Included patients illustrated postoperatively CS of 58 points, VAS of 2.3, complication rate of 18%, FF of 123° and ABD of 112° [49]. All of these studies suggested overall good CS, an acceptable postoperative VAS, a tolerable complication rate and a reasonable ROM in terms of FF and ABD, corresponding with the results of the present study, which has among the included studies the longest follow-up with a mean period of eight years. This suggests, that regardless of implant design, acceptable clinical function can be expected beyond short-term follow-up and throughout a longer period of time. Limitations to good function are mainly due to falls of patients that lead to periprosthetic fractures.

Our data shows that in 78.6% of patients with aseptic loosening (11/14) a complete exchange was necessary because the implanted HA stem design did not allow for conversion to RTSA. This has resulted in an intraoperative humerus fracture during stem removal in 36.3% of cases (4/11). This suggests that more than every third stem removal bears the risk of fracture in the humerus, which then prolongs surgical time and needs additional cable wire fixation. This stresses how important convertible implants are when using a fracture HA in the young to avoid those unnecessary complication.

This study has several limitations. The design is retrospective and the rate of lost-to-follow-up is high due to patients’ old age, their comorbidities and the studys’ long-term follow-up. Due to the lack of certain digitalized information, it was not possible to determine all the details of the patients who could not be followed up, which may affect the overall complication rate in this study. Furthermore, preoperative SSV, VAS for pain, CS, SF-12 and ROM were not assessed, which reduces the ability to comment on procedure related improvement in functional scores. Although good outcomes were illustrated for this cohort, we could not identify prognostic factors that lead to better or poorer outcome due to its small sample size and lack of a control group. In addition, multiple RTSA implants were used and therefore the effect of specific implant design was not evaluated. However, the use of multiple designs and treatment by four different surgeons could be argued as strength to show that acceptable results can be achieved with RTSA regardless of its specific design when implanted accordingly to their technical manual. Lastly, the long follow-up period is a notable strength of this investigation, providing valuable insights into the mid- to long-term outcomes of RTSA after failed initial HA for PHFs.

## Conclusion

In summary, the results of revision RTSA show promising clinical outcomes in patients following a failed HA for fracture treatment. Despite a 15% complication rate leading to revision, functional outcomes are considered acceptable given the satisfactory functional and psychological improvement observed.

## Data Availability

All data generated or analysed during this study are included in this published article.

## Abbreviations

ABD: Abduction
CS: Constant Score
ER: Extrenal Rotation
FF: Forward flexion
HA: Hemiarthroplasty
IR: Internal Rotation
PJI: Periprosthetic joint infection
PCS: Physical component summary
PHF: Proximal humerus fracture
MCS: Mental component summary
ROM: Range of motion
RTSA: Reverse total shoulder arthroplasty
SF-12: Short Form Survey
SSV: Subjective shoulder value
VAS: Visual analogue score
